# Donkey Colostrum and Milk: How Dietary Probiotics Can Affect Metabolomic Profile, Alkaline Sphingomyelinase and Alkaline Phosphatase Activity

**DOI:** 10.3390/metabo13050622

**Published:** 2023-04-30

**Authors:** Fulvio Laus, Luca Laghi, Marilena Bazzano, Maria Grazia Cifone, Benedetta Cinque, Yaosen Yang, Andrea Marchegiani

**Affiliations:** 1 School of Biosciences and Veterinary Medicine, University of Camerino, 62024 Matelica, Italy; fulvio.laus@unicam.it (F.L.); andrea.marchegiani@unicam.it (A.M.); 2 Centre of Foodomics, Department of Agro-Food Science and Technology, University of Bologna, 47521 Cesena, Italy; l.laghi@unibo.it; 3 Department of Life, Health & Environmental Sciences, University of L’Aquila, 67100 L’Aquila, Italy; mariagrazia.cifone@univaq.it (M.G.C.); benedetta.cinque@univaq.it (B.C.)

**Keywords:** donkey milk, metabolomic analysis, probiotics

## Abstract

Positive results on animal health, feed efficiency, and milk’s nutritional content have been obtained after oral administration of probiotics. The aim of the present study was therefore to evaluate the effect of dietary supplementation with high numbers of multispecies probiotic formulations on the milk metabolomic profiles of alkaline sphingomyelinase (alk-SMase) and alkaline phosphatase (ALP) in donkeys. Twenty animals were randomly allocated to receive either a normal diet (group B) or a supplemented diet (group A). Colostrum and milk samples were obtained within 48 h, at 15 days (supplementation start), and at 45 days after parturition. Different metabolomic profiles were observed between colostrum and milk, as were the concentrations of 12 metabolites that changed following 30 days of probiotic supplementation. Alk-SMase activity was found to be higher in donkey colostrum (vs. milk at 15 days); this enzyme, together with ALP, increased in milk after 30 days of probiotic supplementation. The results of the present study provide new insight into the complex changes in donkey colostrum and milk composition in the first 45 days of lactation and how the milk metabolome can be modulated by probiotic supplementation.

## 1. Introduction

Donkey milk, thanks to its compositional similarity to human milk [1], represents a suitable alternative to breastfeeding when the latter is not possible or allowed [2]. Various studies have been devoted to the composition of donkey milk, evidencing its nutraceutical properties [3] and its richness in calcium, selenium [4], and unsaturated fatty acids, especially linoleic acid, while being low in fats and cholesterol. A wide range of positive effects are related to its composition and have been linked to donkey milk consumption. In fact, it is rich in various protective proteins (α-lactalbumin, lysozyme, lactoferrin, lactoperoxidase, and immunoglobulins) and possesses a fatty-acid profile that awards antibacterial, antiviral, antifungal, hypoglycemic, antiparasitic, and antitumor activity [5,6]. Donkey milk is considered a functional food that exerts anti-inflammatory and antioxidative properties with potential positive effects on the delay of aging processes [7], can modulate the immune system [8], and has antimicrobial [9] and possibly anticancer activity [10]. In addition, it does not exacerbate milk intolerance, thanks to its high tolerability [11].

From a nutritional standpoint, milk has traditionally been viewed as a colloidal fluid able to deliver specific health-promoting groups of molecules, such as those mentioned above. This simplistic approach also concerned the assessment of its beneficial effects, generally tested by in-vitro bioassays and on a molecule-by-molecule basis.

In recent years, the advent of omics techniques has opened a window on the compositional complexity of milk, which reflects the complexity of its biosynthesis [12]. In this respect, metabolomics has played the most important role, being the best representation of any food’s phenotype because it is downstream of the genome, transcriptome, and proteome [13,14].

Metabolomics has been applied mainly to cows’ milk by evaluating a variety of factors, both linked to the animal’s genetics and health and to external factors such as husbandry practices. The work of Melzer et al. [15], for example, assessed by metabolomics the relationships between single metabolite concentrations and milk quality traits, while Lu et al. [16] evaluated the effects of dry period, energy balance, and other animal factors on the milk metabolome. In addition, O’Callaghan [17] evaluated how the grazing system reflected on milk’s metabolome. Sheep and goat milks have also been investigated from key perspectives through a metabolomics approach, as can be seen from the works of Scano et al. [18] and Manis et al. [19]. In contrast, donkey’s milk seems underrepresented in the metabolomics literature published so far, despite the promising work from Martini et al., describing the composition of donkey’s milk in response to animal factors [20], or the work by Mecocci et al. [21], where the anti-inflammatory potential of extracellular vesicles has been evaluated.

The constant increase in the global population, the requirements for sustainable animal production that is respectful of animal wellbeing, as well as climate change and the loss of cultivable land, have exacerbated the urgent need for innovative approaches to enhance dairy livestock health and productivity [22]. To address such demand, the use of probiotic supplementation to improve milk production and quality has been tested. As in humans, dietary supplementation with probiotics is a strategy frequently used in farm animal medicine to improve health status through different pathways [23]. Specifically for dairy cows, recent studies reported that Lactobacilli strains administered orally could significantly compete with S. aureus and other mastitis-causing bacteria for adhesion to intestinal epithelium sites [24] and, in turn, to mammary epithelial cells [25], thus hindering the invasion of the mammary gland. It is not unexpected, therefore, that the use of probiotics as additives was found in several studies to have a direct positive impact on feed efficiency and, from there, milk’s yield and milk’s nutrient content [26,27,28].

Additional positive results on animal health have been obtained after oral administration of probiotics to trotter horses [29,30].

Sphingolipids, of which sphingomyelin (SM) is the most abundant in plasma lipoproteins, are structural and functional bioactive lipids found in different foods, including milk, that can act as chemo-protective agents regulating cell growth, differentiation, and death [31]. In fact, once hydrolyzed, SM produces other bioactive molecules, such as ceramide and sphingosine, that play key roles in the maintenance of intestinal mucosal integrity and the inhibition of colon tumorigenesis [31,32]. The alkaline sphingomyelinase (alk-SMase) is a member of the ectonucleotide pyrophosphatase/phosphodiesterase (NPP) family [33]. It is expressed in the intestinal tract and biliary epithelium and is responsible for the digestion of SM [34]. In humans, patients with colorectal adenocarcinoma or ulcerative colitis showed reduced enzymatic activity and protein levels in fecal samples [35,36]. Previous studies have demonstrated that treatment with a multi-strain, high-concentration probiotic formulation led to a significant upregulation of mucosal alk-SMase levels in in vivo models of ulcerative colitis [36]. Significant levels of alk-SMase were also found in the meconium of both preterm and term human infants [37], thus suggesting the natural capacity of newborn mammals to digest SM in the breast milk after birth [38].

Alkaline phosphatase (ALP) is an enzyme naturally found in mammals’ milk that can serve as an indicator of milk pasteurization effectiveness [39]. The milk of all mammals contains ALP, but the levels can be different among species and among animals. In particular, higher ALP activity is detected in sheep, bovines, and goats, while the species with reduced activity are horses, donkeys, and camels. The current validated methods to assess ALP activity in milk are not applicable to all types of milk, and for this reason, the European Food Safety Authority (EFSA) has recently reported the need to evaluate possible alternative methods or indicators for milk produced by animal species with very low ALP activity [40]. The experimentally measured ALP activity in equid milk (equine and donkey) is much lower as compared to other types of milk, such as sheep or bovine [41,42].

The aim of the present study was therefore to evaluate the effect of dietary supplementation with a high concentration of multispecies probiotic formulation (Slab51^®^; Mendes S.A. Lugano, Switzerland) on the milk metabolomic profile in dairy donkeys. Considering that treatment with a multi-strain, high-concentration probiotic formulation was found to upregulate alk-SMase levels in intestinal mucosa [36], a second aim of the study was to assess the alk-SMase activity in the milk samples from donkeys treated with the probiotic formulation (Slab51^®^) as compared with untreated controls. A third aim consisted of testing if the probiotic supplementation was able to increase the ALP enzymatic activity in donkey milk, normally not expressed or expressed below the detection limit, for possible use as a pasteurization process validation marker.

## 2. Materials and Methods

### 2.1. Animals

The study was conducted on 20 clinically healthy Ragusana breed jennies reared for milk production in Italy. Donkeys were housed in individual straw-bedded boxes during the first week after foaling, and thereafter they were moved to common paddocks shared with other jennies and their foals. All the animals lived in the same environment and were fed with about 6 kg/day of polyphyte hay and 0.5 kg/day of concentrates; water was provided ad libitum.

For the scope of the study, jennies were randomly allocated to receive a normal diet (group B control; age 5–10 years; body condition score [43] 2–3 out of 5) or a diet supplemented with probiotics (group A supplemented; age 5–9 years; body condition score 2–3 out of 5) [43].

As inclusion criteria, all the jennies delivered healthy foals, and both jennies and their foals stayed healthy throughout the study. The health status of the animals was checked by clinical examination and routine blood work.

Starting from the 15th day of lactation, each subject included in group A received a single daily dose (18 g) of probiotic Slab51 (SivoMixx^®^, Ormendes SA, Jouxtens-Mézery, CH) for 30 days (Table 1).

Colostrum and milk sampling were performed by manual milking after gentle cleaning of the udder with warm water and drying with a paper towel. All samples were collected in the morning (09.00 a.m.–10.00 a.m.) at the following time points: within 48 h from foaling (colostrum, C), after 15 days of lactation (milk, M15, start of probiotic supplementation), and after 30 days of supplementation (M45, end of probiotic supplementation). All samples were cooled to 5 °C immediately after collection and stored for 2 h at −20 °C until analysis.

All animal-related procedures were in compliance with European Directive 2010/63/EU on the protection of animals used for scientific purposes and approved by the Internal Animal Welfare Committee (approval number 5/2021).

### 2.2. Metabolomic Analysis

Milk samples were centrifuged in sterile tubes (10 min at 1000 g) (Universal 32, Hettich Zentrifugen, Tuttlingen, Germany), and 2 mL aliquots of the supernatant were stored at −20 °C until metabolomic analysis.

The procedure utilized for 1H-NMR analysis was adapted for milk from the one described by Gur for urine [44]. Briefly, a stock solution composed of 3-(trimethylsilyl)-propionic-2,2,3,3-d4 acid sodium salt (TSP) 10 mmol/L and NaN3 2 mmol/L in D2O was obtained. The former served as the NMR spectra’s chemical-shift reference, while the latter avoided bacterial proliferation. The solution was set to a pH of 7.00 ± 0.02 by phosphate buffer (1 M). Milk samples were prepared for 1H-NMR by thawing and centrifuging 1 mL of each sample at 4 °C for 15 min at 18,630 g. The supernatant (700 μL) was added to 100 μL of the NMR analysis solution and centrifuged again.

The spectra were recorded with an AVANCE III spectrometer (Bruker, Milan, Italy), controlled by the Topspin software (Ver. 3.5), at a frequency of 600.13 MHz and a temperature of 298 K. The residual signal from the water was suppressed by pre-saturation, while broad signals from large molecules were reduced by a CPMG filter, set as outlined by Zhu et al. (Zhu 2018). Each spectrum was acquired by summing up 256 transients, registering 32 K data points over a 7184 Hz spectral window, with an acquisition time of 2.28 s and a relaxation delay of 5 s. In Topspin, a manual correction phase was applied to each spectrum, together with a line-broadening of 0.3 Hz. Signal assignment was performed by comparing their chemical shift and multiplicity with the Human Metabolome Database (Wishart 2007) and the Chenomx software library (Chenomx Inc., Edmonton, AB, Canada, v10) by means of Chenomx software routines. The subsequent steps were performed in the R computational language by means of scripts developed in-house. The absolute concentration of molecules was measured in the sample with the median water dilution, assessed by probabilistic quotient normalization (PQN) [45]. For this purpose, TSP was used as an internal standard. The concentration of each molecule was obtained from the area of one of its signals, calculated by the GSD (global spectra deconvolution) algorithm, implemented in MestReNova software (Mestrelab research S.L. Santiago De Compostela (Spain)—ver 14.2.0-26256), by considering an LOQ (limit of quantification) of 5. This occurred after applying a baseline adjustment by the Whittaker Smoother procedure and a line-broadening of 0.3 Hz. Differences in water content between the reference sample and any other were compensated by PQN.

### 2.3. Alkaline Sphingomyelinase Assay

Alkaline sphingomyelinase (alk-SMase) enzymatic activity was assayed according to the previously reported method [46] and slightly modified. For each sample, 50 microliters were resuspended in an alkaline buffer solution containing 50 mM of tris-hydrochloride, 0.15 M of sodium chloride, 2 mM EDTA (pH 9.0), and a 3 mM bile salt mixture of taurocholic acid, taurodeoxycholic acid, glycocholic acid, and glycochenodeoxycholic acid, with a molar ratio of 3:2:1.8:1. The enzyme reaction was started by the addition of 10 nmol of C12-NBD Sphingomyelin (N-[12-[(7-nitro-2-1,3-benzoxadiazol-4-yl)amino]dodecanoyl]-sphingosine-1-phosphocholine Avanti Polar Lipids, Inc, Alabaster, Alabama) in the alkaline reaction buffer in a total volume of 200 µL. After incubation at 37 °C for 1 h, the reaction was stopped by the addition of 200 µL chloroform:methanol (2:1, *v*/*v*), the samples were centrifuged for 10 min at 22,000× *g*, the organic phases were extracted, and, at the aqueous phases, 400 µL chloroform:methanol (2:1, *v*/*v*) were added. The samples were centrifuged, and the organic phases were added to the organic lipid phases previously obtained. The organic lipid phases were evaporated under a N2 stream and then dissolved in 80 µL of chloroform. The samples were spotted onto a thin-layer chromatography (TLC) plate (Merck, Kenilworth, NJ, USA) and separated with chloroform:methanol:water (65:25:4, *v*/*v*/*v*) being used as a solvent. Under these conditions, NBD-ceramide appeared as a single spot. The emission intensities of the fluorescent ceramide spots were determined by UVItec Alliance (Cambridge, UK). Densitometric analysis was performed by software that was provided by the company. The amounts of pmol ceramide generated from NBD-SM by alk-SMase activity were obtained by interpolating the respective fluorescence intensities in the calibration plot of C12-NBD Ceramide (N-[12-[(7-nitro-2-1,3-benzoxadiazol-4-yl)amino]dodecanoyl]-D-erythro-sphingosine, Avanti Polar Lipids, Inc, Alabaster, Alabama) concentration vs. fluorescence intensity. The alk-SMase activity was expressed as picomoles of ceramide produced per h/mL.

### 2.4. Photometric Alkaline Phosphatase Assay

ALP activity was measured by a colorimetric alkaline phosphatase assay kit (Abcam, Cambridge, UK). The reagents and samples were prepared according to the manufacturer’s instructions. The absorbance was measured by spectrophotometric reading at 405 nm using a microplate reader (Bio-Rad Laboratories, Milan, Italy). One unit (U) of ALP activity is defined as the amount of enzyme that catalyzes the transformation of one micromole of p-nitrophenyl phosphate (pNPP) per minute under standard assay conditions.

### 2.5. Statistical Analysis

Statistical analysis was performed using GraphPad Prism 8 (GraphPad Software Inc., Boston, MA, USA). A student *t* test was run to highlight statistical differences between colostrum C and mature milk M15 and between the supplemented and control groups after 30 days of supplementation M45, considering significant values of *p* < 0.05.

## 3. Results

Metabolomic analysis of donkey colostrum and milk identified 76 metabolites by 1H-NMR spectra, including sugars, amino acids and derivatives, energy metabolites, fatty acids and associated metabolites, nucleotides and derivatives, and others (Table 2).

**Figure 1:**
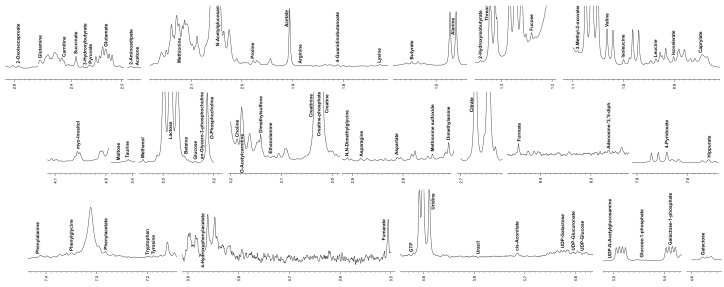
Representative spectra of donkey colostrum.

Figure 1 shows representative spectra obtained for donkey colostrum. Exact quantitation of 14 metabolites, including adenosine-3,5-diphosphate, phenylalanine, tryptophan, cytidine, orotate, arginine, uracil, lysine, 3-methyl-2-oxovalerate, 3-hydroxybutyrate, ethanolamine, UDP-galactose, UDP-glucuronate, and UDP-N-acetylglucosamine, was difficult due to their low concentrations or severe spectral overlap and was thus not reported. The comparison of milk and colostrum metabolomic profiles highlighted significant differences in content for 18 metabolites (*p* < 0.05). Namely, galactose, galactose-1-phosphate, fumarate, uridine, dimethyl sulphone, creatine phosphate, sn glycerol-3-phosphocholine, o-phosphocholine, o-acetylcarnitine, and ethanol decreased in mature milk compared to colostrum. Conversely, myoinositol, creatine, acetone, alanine, betaine, valine, glutamate, and caprylate were found at higher concentrations in milk compared to colostrum.

Different concentrations (*p* < 0.05) of 12 metabolites were observed in milk from the experimental group after 30 days of probiotic supplementation compared to controls. Namely, lactose, O-phosphocholine, sn-Glycero-3-phosphocholine, and 4-pyridoxate were higher in the supplemented group, whereas caprylate, isovalerate, butyrate, 2-oxoisocaproate, glucose, glucose-1-phosphate, glutamine, and 4-hydroxyphenilacetate were lower compared to the control group (Table 3).

### 3.1. Alkaline Sphingomyelinase Activity in Colostrum and Milk

**Figure 2:**
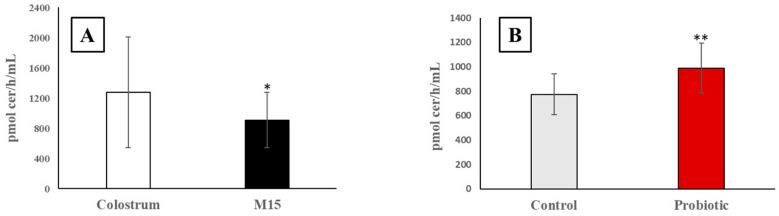
Alkaline sphingomyelinase (alk-SMase) activity detected in colostrum vs. milk at 15 days (M15) (**A**) and in milk from control vs. probiotic supplemented donkeys (**B**). Mean values and standard deviations of alk-SMase are expressed as picomoles of ceramide produced per h/mL. * significance (*p* < 0.05) vs. colostrum; ** significance (*p* < 0.05) vs. control.

Alk-SMase activity, expressed as pmoles ceramide/h/mL, was first compared between colostrum and milk samples collected from 20 donkeys at 48 h and 15 days, respectively (Figure 2A). The enzymatic activity of the colostrum samples was significantly higher compared to milk collected after 15 days of lactation (M15). Of note, the probiotic supplementation for 30 days caused a moderate but statistically significant increment of alk-SMase activity in the milk samples compared to untreated ones (Figure 2B).

### 3.2. Alkaline Phosphate Activity in Colostrum and Milk

The ALP activity was not detected by photometric assay in colostrum or mature milk at 15 and 45 days, respectively. Of note, the treatment with the probiotic formulation increased the activity to detectable levels in six out of ten animals (Table 4).

## 4. Discussion

The results of the present study provide new insight into the complex changes of the donkey milk metabolome in the first 45 days of lactation and provide evidence that this can be modulated by dietary supplementation. Few studies investigated the milk metabolome in equids, focusing on their nutritional properties [47,48], and no study dealt with the individual colostrum and milk metabolomic profiles in equids from a clinical point of view.

Donkey milk is becoming increasingly popular as a natural alternative milk for various categories of consumers, especially infants and the elderly population, and this has deepened knowledge on milk production and composition [49]. The latter is influenced by many factors, such as breed, farm management, feeding, stage and number of lactations, foaling season, and milking procedure [50]. A recent study investigated the detailed chemical composition of milk, including macro- and micro-mineral elements, of the Ragusano donkey reared on a specialized dairy farm from the second to the ninth month of lactation [51]. The data herein presented contribute to a clean depiction of Ragusano milk composition over lactation and further underline a couple of main aspects: (i) a standardized nutritional profile of donkey milk needs to be further investigated on a breed-oriented basis to provide more conclusive insights about the composition of milk; and (ii) the nutritional properties of donkey milk and its potential derivatives may be modulated by dietary supplementation.

To the best of the authors’ knowledge, the only paper investigating the metabolomic composition of donkey milk applied an untargeted metabolomic approach using bulk tank milk [1,48], but the possible influence of dietary supplementation has not been investigated before the current investigation.

Comparing the metabolomes of colostrum and milk, the most relevant change with clinical relevance is the increase in acetone in milk compared to colostrum, which can be used for monitoring the health status of the dam. In cows, milk acetone level is widely used as a biomarker of subclinical ketosis, milk yield, and reproductive efficiency [52]. Increasing levels of ketone bodies in milk after parturition could be indicative of a negative energy balance that is common in dairy animals, particularly in the early stages of lactation, as milk yield increases dramatically at the onset of lactation and consumption of food to meet these requirements can be limited [52].

A further difference between milk and colostrum concerns increased levels (in milk) of metabolites involved in energetic metabolism such as galactose, galactose-1-phosphate, fumarate, creatine-phosphate, sn-Glycero-3-phosphocholine, O-Phosphocholine, O-Acetylcarnitine, urydine, and dimethyl sulphone. Changes observed in such metabolites are similar to previous results in dairy cows, where glucose and galactose were higher in colostrum and decreased in mature milk. This evidence could be explained as the main milk carbohydrate, lactose, is synthesized from free glucose and galactose [53]. The monitoring of this metabolite in milk has clinical relevance in dairy cows, as it was found that lactose levels are related to udder health, energy balance, and metabolism [54].

Betaine, also known as trimethylglycine, is an aminoacid synthesized through choline metabolism, in which it acts as a methyl group donor to the toxic metabolite homocysteine, converting it to methionine [55]. Betaine possesses essential biochemical functions: (i) being an osmolyte, it supports the maintenance of the intracellular osmotic pressure similar to other electrolytes and stabilizes both protein structure and function, thus protecting cells, proteins, and enzymes from osmotic stress; (ii) it is able to protect the liver from steatosis and maintain intestinal epithelial barrier integrity; and (iii) maternal betaine supplementation normalizes fetal growth and adiposity of progeny in an experimental model by reducing glucose and fatty acid transporters and the growth-promoting insulin-like growth factor 2 in the placenta [55]. In cows, betaine has been supplemented to improve production performance and protect cows from heat-related oxidative stress [56].

Glycerophosphocholine and phosphocholine are major choline metabolites in milk that are necessary in neonates for rapid organ growth and membrane biosynthesis [57].

Dimethyl sulfone is an organic sulfur-containing compound that occurs naturally in animals, including humans and a variety of fruits, vegetables, and grains [58]. It has been shown to possess anti-inflammatory effects in a murine model of inflammation, and its presence in milk may suggest a possible anti-inflammatory role for donkey milk, as very recently suggested [21].

In the present study, 15 days after parturition, the jennies’ diet was supplemented with probiotics, namely a mixture of lactic acid bacteria and bifidobacteria (SLAB51 formulation) for 30 days. Similar studies have been conducted on dairy cows, in which supplementation has been extensively studied as a strategy to improve the nutritional quality of cow milk as well as boost growth and health in dairy calves [59,60].

According to our results, the milk metabolome of animals receiving the probiotic supplement was characterized by higher levels of lactose, O-phosphocholine, and 4-pyridoxate and a lower content of metabolites, including glucose-1-P, caprylate, isovalerate, butyrate, 4-hydroxyphenylacetate, and 2-oxoisocaproate.

Higher levels of lactose in milk exert a direct influence on milk yield as it represents the main osmotic regulator between the blood and alveolar lumen. Lactose affects the amount of absorbed water in the alveoli and, thus, the volume of produced milk [61].

Butyrate is a four-carbon short-chain fatty acid produced by microbial fermentation of dietary fibers in the lower intestinal tract and absorbed by colonocytes. Recently, butyrate has received specific attention for its beneficial effects on intestinal homeostasis and energy metabolism [62]. With its anti-inflammatory properties, butyrate acts as a modulator of chemotaxis and adhesion of intestinal immune cells, enhancing intestinal barrier function and mucosal immunity [63,64]. Moreover, betaine butyrate plays a role in the maintenance of the gut-brain axis and related homeostasis [65].

Lactation is an energy-consuming process that reduces glutamine milk concentration over time [66], as observed in the present study. Glutamine is an essential amino acid that plays a central role in milk production [66], and it has been extensively studied in dairy animals, where ruminants have a relatively low glutamine synthetase capacity compared with monogastric species [67]. Glutamine is quantitatively the most abundant free amino acid in milk and the most important energy source for the intestinal tissue of newborns [68]. It has crucial roles for the promotion and maintenance of cell functions (i.e., glutamate–glutamine cycle, proline synthesis), and it has recently been discovered that glutamine is essential to overcome metabolic stress [69,70]. In ruminants, it has been proposed as a limiting factor in milk quality and production, but the debate is still open.

Thirty days of SLAB51 probiotic supplementation were able to lessen the deleterious effect of lactation on milk glutamine content. In fact, supplemented animals showed a reduced decrease in glutamine and proline concentrations when compared to control animals. The same trend was registered for proline [67].

Isovalerate is a branched-chain saturated fatty acid anion that is the conjugate base of isovaleric acid. It has been reported to improve ruminal fermentation, rumen leucine production (https://pubchem.ncbi.nlm.nih.gov/compound/Isovalerate; accessed on 1 March 2023) and feed digestion in cattle [71]. It has a role as a mammalian and plant metabolite. In dairy calves, isovalerate supplementation was shown to promote the development of small intestinal mucosa in a dose-dependent manner [65]. 3-Hydroxyisovaleric acid (3HIA), a form of isovaleric acid, is an alternative metabolite in the pathway of leucine catabolism and can serve as an indicator of energy status in dairy cows [72], but its role in donkey milk has not been elucidated.

As for previous published studies, the present highlights that donkey milk composition is not standardized, and a high degree of variability is reported in the restricted literature. Our study considered only the Ragusano breed, so the results provided should be referred only to this breed, as both genetic and environmental factors play important roles in milk composition.

All the metabolites found to be influenced by probiotic supplementation in both donkey colostrum and milk exert known and specified biological roles in human health. As a limitation in the discussion of the result of the present study, not all metabolites found have been deeply analyzed yet in veterinary medicine, and their biochemical and/or clinical role has not been ascertained yet; thus, it was not possible to speculate on a possible clinical implication. We hope the results obtained in this paper may open new research routes aimed at the analysis of the complex and fascinating metabolome of donkey milk.

SLAB51 supplementation in the jennies led to a significant enrichment of the alk-SMase level in the milk samples. This is, to the best of the authors’ knowledge, the first attempt to assess the activity of alk-SMase in milk. For end users, this effect can lead to an improvement of mucosal intestinal homeostasis due to the ability of this enzyme to generate phosphocholine and ceramide, inactivate platelet-activating factor (PAF), and counteract the release of lysophosphatidic acid (LPA). These properties are associated with anti-inflammatory and anti-tumorigenic effects [70,73]. Moreover, alk-SMase, expressed in the gut of preterm and term newborn infants, has been implicated in differentiating the gut epithelium [37]. Then, infants fed donkey milk enriched with a probiotic able to furnish appropriate levels of active alk-SMase could guarantee full physiological intestinal development.

In the present study, we have also considered the ALP activity variation over time and supplementation. ALP activity is the most widely used indicator of pasteurization effectiveness in milk, and its reduction after thermal treatment permits us to consider the product safe from a microbiological point of view. When the ALP is undetectable, as normally happens in donkey milk, it cannot be used as an indicator [74]. The SLAB51 supplementation was able to increase the levels of ALP over the threshold detection limit in six out of ten donkeys, laying the basis for possible further studies aimed at using ALP as a marker for the assessment of efficient pasteurization in donkey milk.

## 5. Conclusions

The findings herein presented the different metabolomic compositions between colostrum and milk and proved that dietary probiotics can modulate milk composition, opening the doors to the exciting new age of dietary supplementation for modulation in dairy donkeys and its application for human consumption.

## Figures and Tables

**Table 1:** Bacterial content (in the colony forming unit, CFU) of each strain administered daily for 30 days to the jennies of the supplemented group (A).

Bacterial Strain in SLAB51^®^ Blend	Total Number of CFU for 18 g
*Streptococcus thermophilus* DSM 32245/CNCM I-5570	960 Billion
*Lactobacillus brevis* DSM 27961/CNCM I-5566	432 Billion
*Bifidobacterium lactis* DSM 32246/CNCM I-5571	300 Billion
*Bifidobacterium lactis* DSM 32247/CNCM I-5572	300 Billion
*Lactobacillus plantarum* DSM 32244/CNCM I-5569	192 Billion
*Lactobacillus paracasei* DSM 32243/CNCM I-5568	144 Billion
*Lactobacillus acidophilus* DSM 32241/CNCM I-5567	60 Billion
*Lactobacillus helveticus* DSM 32242/CNCM I-5573	12 Billion

**Table 2:** Metabolites found in donkey colostrum and milk. Values are expressed in mmol/L. In bold are metabolites whose concentrations significantly differ between colostrum and milk.

Molecule (mmol/L)	Colostrum (C)	Milk (M15)	*p* Values
Formate	0.017 ± 0.006	0.014 ± 0.003	0.102
4-Pyridoxate	0.176 ± 0.101	0.189 ± 0.078	0.488
Hippurate	0.172 ± 0.107	0.172 ± 0.067	0.992
Phenylglycine	0.010 ± 0.011	0.048 ± 0.108	0.123
Phenylacetate	0.037 ± 0.020	0.035 ± 0.012	0.709
Tyrosine	0.026 ± 0.009	0.026 ± 0.005	0.906
4-Hydroxyphenylacetate	0.028 ± 0.008	0.032 ± 0.009	0.181
Fumarate	0.011 ± 0.004	0.008 ± 0.002	0.002
Uridine	0.565 ± 0.322	0.187 ± 0.190	0.000
cis-Aconitate	0.034 ± 0.018	0.027 ± 0.005	0.163
UDP-glucose	0.084 ± 0.084	0.061 ± 0.011	0.241
Glucose-1-phosphate	0.128 ± 0.074	0.089 ± 0.094	0.111
Galactose-1-phosphate	1.635 ± 0.407	1.251 ± 0.385	0.007
Galactose	0.888 ± 0.096	0.801 ± 0.134	0.018
myo-Inositol	1.226 ± 0.518	1.780 ± 0.594	0.006
Maltose	0.130 ± 0.058	0.136 ± 0.038	0.653
Taurine	1.004 ± 0.237	1.026 ± 0.541	0.855
Methanol	0.047 ± 0.019	0.052 ± 0.041	0.617
Lactose	162.428 ± 33.321	165.190 ± 33.996	0.725
Betaine	0.032 ± 0.025	0.063 ± 0.066	0.035
TMAO	0.026 ± 0.016	0.020 ± 0.012	0.173
Glucose	0.689 ± 0.218	0.673 ± 0.219	0.802
sn-Glycero-3-phosphocholine	1.818 ± 0.546	1.450 ± 0.410	0.029
O-Phosphocholine	0.245 ± 0.183	0.148 ± 0.171	0.045
Choline	0.035 ± 0.051	0.032 ± 0.048	0.845
O-Acetylcarnitine	0.080 ± 0.037	0.056 ± 0.020	0.017
Dimethyl sulfone	0.027 ± 0.010	0.017 ± 0.007	0.003
Malonate	0.011 ± 0.005	0.013 ± 0.006	0.182
Creatinine	0.087 ± 0.071	0.066 ± 0.040	0.155
Creatine-phosphate	1.259 ± 0.437	0.920 ± 0.400	0.023
Creatine	0.637 ± 0.148	0.724 ± 0.215	0.030
N.N-Dimethylglycine	0.003 ± 0.001	0.003 ± 0.000	0.053
Asparagine	0.075 ± 0.026	0.089 ± 0.022	0.110
Aspartate	0.631 ± 1.027	0.262 ± 0.262	0.133
Methionine sulfoxide	0.007 ± 0.002	0.006 ± 0.001	0.056
Dimethylamine	0.007 ± 0.003	0.008 ± 0.003	0.239
Citrate	5.662 ± 2.117	5.284 ± 1.435	0.517
2-Oxoisocaproate	0.039 ± 0.029	0.047 ± 0.022	0.272
Glutamine	0.804 ± 0.881	1.069 ± 0.792	0.347
Carnitine	0.154 ± 0.055	0.130 ± 0.066	0.238
Succinate	0.074 ± 0.024	0.066 ± 0.011	0.161
Pyruvate	0.016 ± 0.012	0.021 ± 0.019	0.334
Glutamate	0.757 ± 0.354	1.144 ± 0.339	0.000
2-Aminoadipate	0.037 ± 0.020	0.037 ± 0.013	0.930
Acetone	0.004 ± 0.001	0.005 ± 0.001	0.005
Methionine	0.127 ± 0.108	0.153 ± 0.136	0.534
N-Acetylglucosamine	0.273 ± 0.488	0.208 ± 0.247	0.565
Proline	0.233 ± 0.115	0.226 ± 0.100	0.839
Acetate	0.134 ± 0.072	0.136 ± 0.078	0.924
4-Guanidinobutanoate	0.039 ± 0.011	0.047 ± 0.016	0.079
Butyrate	0.051 ± 0.095	0.085 ± 0.077	0.182
Alanine	0.202 ± 0.072	0.263 ± 0.087	0.011
2-Hydroxyisobutyrate	0.004 ± 0.002	0.003 ± 0.001	0.051
Threonine	0.383 ± 0.333	0.730 ± 1.062	0.173
Lactate	0.775 ± 1.544	1.672 ± 4.603	0.410
Fucose	0.081 ± 0.030	0.086 ± 0.055	0.736
Ethanol	286.591 ± 46.471	261.203 ± 43.007	0.042
Valine	0.110 ± 0.063	0.148 ± 0.050	0.033
Isoleucine	0.028 ± 0.028	0.043 ± 0.022	0.079
Leucine	0.035 ± 0.028	0.041 ± 0.023	0.491
Isovalerate	0.038 ± 0.025	0.039 ± 0.019	0.965
Caprylate	0.271 ± 0.185	0.635 ± 0.484	0.004
TSP	6.460 ± 2.342	6.088 ± 1.282	0.506

**Table 3:** Metabolites found in the milk of the experimental and control groups: values are expressed in mmol/L. In bold are metabolites whose concentrations significantly differ after probiotic supplementation.

Molecule (mmol/L)	Group B (Supplemented)	Group A (Control)	*p* Values
Formate	0.016 ± 0.004	0.014 ± 0.002	0.075
4-Pyridoxate	0.183 ± 0.071	0.267 ± 0.032	0.001
Hippurate	0.164 ± 0.043	0.161 ± 0.102	0.465
Phenylglycine	0.017 ± 0.017	0.009 ± 0.008	0.092
Phenylacetate	0.036 ± 0.008	0.033 ± 0.007	0.182
Tyrosine	0.026 ± 0.003	0.030 ± 0.009	0.118
4-Hydroxyphenylacetate	0.033 ± 0.007	0.024 ± 0.009	0.010
Fumarate	0.009 ± 0.002	0.008 ± 0.002	0.101
Uridine	0.145 ± 0.097	0.139 ± 0.222	0.465
cis-Aconitate	0.032 ± 0.006	0.030 ± 0.006	0.190
UDP-glucose	0.070 ± 0.009	0.065 ± 0.018	0.173
Glucose-1-phosphate	0.082 ± 0.064	0.036 ± 0.025	0.022
Galactose-1-phosphate	1.339 ± 0.150	1.178 ± 0.305	0.067
Galactose	0.888 ± 0.170	0.927 ± 0.142	0.288
myo-Inositol	1.870 ± 0.509	2.048 ± 0.124	0.148
Maltose	0.159 ± 0.046	0.136 ± 0.062	0.176
Taurine	0.794 ± 0.255	0.661 ± 0.180	0.093
Methanol	0.036 ± 0.009	0.047 ± 0.022	0.078
Lactose	177.382 ± 13.310	193.809 ± 9.391	0.002
Betaine	0.042 ± 0.018	0.051 ± 0.008	0.073
TMAO	0.011 ± 0.006	0.016 ± 0.012	0.107
Glucose	0.714 ± 0.170	0.551 ± 0.197	0.028
sn-Glycero-3-phosphocholine	1.345 ± 0.160	1.557 ± 0.329	0.036
O-Phosphocholine	0.068 ± 0.050	0.172 ± 0.150	0.022
Choline	0.014 ± 0.006	0.022 ± 0.015	0.056
O-Acetylcarnitine	0.055 ± 0.018	0.050 ± 0.015	0.232
Dimethyl sulfone	0.015 ± 0.006	0.019 ± 0.008	0.078
Malonate	0.011 ± 0.005	0.012 ± 0.003	0.491
Creatinine	0.050 ± 0.018	0.061 ± 0.018	0.089
Creatine-phosphate	0.892 ± 0.159	0.925 ± 0.218	0.347
Creatine	0.821 ± 0.172	0.766 ± 0.133	0.210
N.N-Dimethylglycine	0.003 ± 0.000	0.003 ± 0.001	0.114
Asparagine	0.093 ± 0.024	0.076 ± 0.030	0.081
Aspartate	0.233 ± 0.213	1.061 ± 2.710	0.162
Methionine sulfoxide	0.006 ± 0.000	0.006 ± 0.001	0.411
Dimethylamine	0.009 ± 0.003	0.007 ± 0.003	0.054
Citrate	4.284 ± 1.180	4.589 ± 1.378	0.296
2-Oxoisocaproate	0.051 ± 0.014	0.030 ± 0.010	0.000
Glutamine	1.255 ± 0.308	0.841 ± 0.541	0.021
Carnitine	0.152 ± 0.119	0.110 ± 0.046	0.154
Succinate	0.061 ± 0.012	0.058 ± 0.014	0.328
Pyruvate	0.014 ± 0.005	0.014 ± 0.002	0.449
Glutamate	1.257 ± 0.207	1.191 ± 0.213	0.243
2-Aminoadipate	0.041 ± 0.015	0.035 ± 0.009	0.143
Acetone	0.005 ± 0.001	0.004 ± 0.001	0.152
Methionine	0.097 ± 0.032	0.110 ± 0.111	0.351
N-Acetylglucosamine	0.119 ± 0.049	0.137 ± 0.119	0.328
Proline	0.162 ± 0.057	0.206 ± 0.095	0.105
Acetate	0.134 ± 0.057	0.117 ± 0.058	0.263
4-Guanidinobutanoate	0.044 ± 0.010	0.041 ± 0.011	0.241
Butyrate	0.201 ± 0.190	0.070 ± 0.046	0.024
Alanine	0.277 ± 0.071	0.228 ± 0.079	0.076
2-Hydroxyisobutyrate	0.004 ± 0.002	0.003 ± 0.001	0.107
Threonine	0.336 ± 0.111	0.393 ± 0.128	0.146
Lactate	0.242 ± 0.083	0.287 ± 0.124	0.167
Fucose	0.114 ± 0.091	0.098 ± 0.034	0.308
Ethanol	268.419 ± 20.585	317.981 ± 20.994	0.000
Valine	0.140 ± 0.029	0.123 ± 0.037	0.131
Isoleucine	0.032 ± 0.012	0.032 ± 0.009	0.482
Leucine	0.030 ± 0.010	0.034 ± 0.011	0.210
Isovalerate	0.045 ± 0.016	0.020 ± 0.011	0.000
Caprylate	0.728 ± 0.342	0.408 ± 0.164	0.007
TSP	6.789 ± 0.468	7.438 ± 0.623	0.007

**Table 4:** Alkaline phosphatase activity. Alkaline phosphatase (ALP) activity was assayed in colostrum, in milk collected after 15 days of lactation, and in milk samples derived from untreated (control group) and treated with probiotic formulation (probiotic group) donkeys for 30 days. The ALP was expressed as U/mL, where one unit (U) of ALP activity is defined as the amount of enzyme that catalyzes the transformation of one micromole of p-nitrophenyl phosphate (pNPP) per minute under standard assay conditions. N.D. is not detectable.

Sample	Colostrum	M15	Milk at 45 Days (Control Group)	Milk at 45 Days (Probiotic Group)
ALP (U/mL)	N.D.	N.D.	N.D.	Range 0.97–11.73

## Data Availability

The data presented are available in this study.

## References

[1] Murgia A., Scano P., Contu M., Ibba I., Altea M., Bussu M., Demuru M., Porcu A., Caboni P. (2016). Characterization of Donkey Milk and Metabolite Profile Comparison with Human Milk and Formula Milk. LWT.

[2] Bertino E., Agosti M., Peila C., Corridori M., Pintus R., Fanos V. (2022). The Donkey Milk in Infant Nutrition. Nutrients.

[3] Martini M., Altomonte I., Licitra R., Salari F. (2018). Nutritional and Nutraceutical Quality of Donkey Milk. J. Equine Vet. Sci..

[4] Li Y., Ma Q.-S., Zhou M.-M., Zhang Z.-W., Zhan Y.-D., Liu G.-Q., Zhu M.-X., Wang C.-F. (2022). A Metabolomics Comparison in Milk from Two Dezhou Donkey Strains. Eur. Food Res. Technol..

[5] Di Salvo E., Conte F., Casciaro M., Gangemi S., Cicero N. (2022). Bioactive Natural Products in Donkey and Camel Milk: A Perspective Review. Nat. Prod. Res..

[6] Živkov Baloš M., Ljubojević Pelić D., Jakšić S., Lazić S. (2023). Donkey Milk: An Overview of Its Chemical Composition and Main Nutritional Properties or Human Health Benefit Properties. J. Equine Vet. Sci..

[7] Li Y., Ma Q., Liu G., Wang C. (2022). Effects of Donkey Milk on Oxidative Stress and Inflammatory Response. J. Food Biochem..

[8] Garhwal R., Sangwan K., Mehra R., Kumar N., Bhardwaj A., Pal Y., Buttar H.S., Kumar H. (2022). A Systematic Review of the Bioactive Components, Nutritional Qualities and Potential Therapeutic Applications of Donkey Milk. J. Equine Vet. Sci..

[9] Zhang X.Y., Zhao L., Jiang L., Dong M.L., Ren F.Z. (2008). The Antimicrobial Activity of Donkey Milk and Its Microflora Changes during Storage. Food Contro..

[10] Mao X., Gu J., Sun Y., Xu S., Zhang X., Yang H., Ren F. (2009). Anti-Proliferative and Anti-Tumour Effect of Active Components in Donkey Milk on A549 Human Lung Cancer Cells. Int. Dairy J..

[11] Polidori P., Vincenzetti S. (2013). Use of Donkey Milk in Children with Cow’s Milk Protein Allergy. Foods.

[12] Sun H.Z., Sun H.Z., Zhou M., Wang O., Chen Y., Liu J.X., Guan L.L. (2020). Multi-Omics Reveals Functional Genomic and Metabolic Mechanisms of Milk Production and Quality in Dairy Cows. Bioinformatics.

[13] Laghi L., Picone G., Capozzi F. (2014). Nuclear Magnetic Resonance for Foodomics beyond Food Analysis. Trends Anal. Chem..

[14] Munekata P.E.S., Pateiro M., Rocchetti G., Domínguez R., Rocha J.M., Lorenzo J.M. (2022). Application of Metabolomics to Decipher the Role of Bioactive Compounds in Plant and Animal Foods. Curr. Opin. Food Sci..

[15] Melzer N., Wittenburg D., Hartwig S., Jakubowski S., Kesting U., Willmitzer L., Lisec J., Reinsch N., Repsilber D. (2013). Investigating Associations between Milk Metabolite Profiles and Milk Traits of Holstein Cows. J. Dairy Sci..

[16] Lu J., Antunes Fernandes E., Páez Cano A.E., Vinitwatanakhun J., Boeren S., Van Hooijdonk T., Van Knegsel A., Vervoort J., Hettinga K.A. (2013). Changes in Milk Proteome and Metabolome Associated with Dry Period Length, Energy Balance, and Lactation Stage in Postparturient Dairy Cows. J. Proteome Res..

[17] O’Callaghan T.F., Vázquez-Fresno R., Serra-Cayuela A., Dong E., Mandal R., Hennessy D., McAuliffe S., Dillon P., Wishart D.S., Stanton C. (2018). Pasture Feeding Changes the Bovine Rumen and Milk Metabolome. Metabolites.

[18] Scano P., Carta P., Ibba I., Manis C., Caboni P. (2020). An Untargeted Metabolomic Comparison of Milk Composition from Sheep Kept Under Different Grazing Systems. Dairy.

[19] Manis C., Scano P., Nudda A., Carta S., Pulina G., Caboni P. (2021). LC-QTOF/MS Untargeted Metabolomics of Sheep Milk under Cocoa Husks Enriched Diet. Dairy.

[20] Martini M., Altomonte I., Manica E., Salari F. (2015). Changes in Donkey Milk Lipids in Relation to Season and Lactation. J. Food Compos. Anal..

[21] Mecocci S., Gevi F., Pietrucci D., Cavinato L., Luly F.R., Pascucci L., Petrini S., Ascenzioni F., Zolla L., Chillemi G. (2020). Anti-Inflammatory Potential of Cow, Donkey and Goat Milk Extracellular Vesicles as Revealed by Metabolomic Profile. Nutrients.

[22] Britt J.H., Cushman R.A., Dechow C.D., Dobson H., Humblot P., Hutjens M.F., Jones G.A., Ruegg P.S., Sheldon I.M., Stevenson J.S. (2018). Invited Review: Learning from the Future-A Vision for Dairy Farms and Cows in 2067. J. Dairy Sci..

[23] Delia E., Tafaj M., Männer K., Delia E., Tafaj M., Männer K. (2012). Efficiency of Probiotics in Farm Animals. Probiotic. Anim..

[24] Pellegrino M.S., Frola I.D., Natanael B., Gobelli D., Nader-Macias M.E.F., Bogni C.I. (2019). In Vitro Characterization of Lactic Acid Bacteria Isolated from Bovine Milk as Potential Probiotic Strains to Prevent Bovine Mastitis. Probiotics Antimicrob Proteins.

[25] Steinberg R.S., de Silva L.C.S., de Souza M.R., Reis R.B., Bicalho A.F., Nunes J.P.S., Dias A.A.M., Nicoli J.R., Neumann E., Nunes C. (2022). Prospecting of Potentially Probiotic Lactic Acid Bacteria from Bovine Mammary Ecosystem: Imminent Partners from Bacteriotherapy against Bovine Mastitis. Int. Microbiol..

[26] Moallem U., Lehrer H., Livshitz L., Zachut M., Yakoby S. (2009). The Effects of Live Yeast Supplementation to Dairy Cows during the Hot Season on Production, Feed Efficiency, and Digestibility. J. Dairy Sci..

[27] Peng H., Wang J.Q., Kang H.Y., Dong S.H., Sun P., Bu D.P., Zhou L.Y. (2012). Effect of Feeding Bacillus Subtilis Natto Fermentation Product on Milk Production and Composition, Blood Metabolites and Rumen Fermentation in Early Lactation Dairy Cows. J. Anim. Physiol. Anim. Nutr..

[28] So S., Wanapat M., Cherdthong A. (2021). Effect of Sugarcane Bagasse as Industrial By-Products Treated with Lactobacillus Casei TH14, Cellulase and Molasses on Feed Utilization, Ruminal Ecology and Milk Production of Mid-Lactating Holstein Friesian Cows. J. Sci. Food Agric..

[29] Laghi L., Zhu C., Campagna G., Rossi G., Bazzano M., Laus F. (2018). Probiotic Supplementation in Trained Trotter Horses: Effect on Blood Clinical Pathology Data and Urine Metabolomic Assessed in Field. J. Appl. Physiol..

[30] Schoster A., Weese J.S., Guardabassi L. (2014). Probiotic Use in Horses—What Is the Evidence for Their Clinical Efficacy?. J. Vet. Intern. Med..

[31] Iqbal J., Walsh M.T., Hammad S.M., Hussain M.M. (2017). Sphingolipids and Lipoproteins in Health and Metabolic Disorders. Trends Endocrinol. Metab..

[32] Marzo F., Jauregui P., Barrenetxe J., Martínez-Peñuela A., Ibañez F.C., Milagro F.I. (2022). Effect of a Diet Supplemented with Sphingomyelin and Probiotics on Colon Cancer Development in Mice. Probiotics Antimicrob. Proteins.

[33] Duan R.D., Bergman T., Xu N., Wu J., Cheng Y., Duan J., Nelander S., Palmberg C., Nilsson Å. (2003). Identification of Human Intestinal Alkaline Sphingomyelinase as a Novel Ecto-Enzyme Related to the Nucleotide Phosphodiesterase Family. J. Biol. Chem..

[34] Duan R.D. (2006). Alkaline Sphingomyelinase: An Old Enzyme with Novel Implications. Biochim. Biophys. Acta.

[35] Di Marzio L., Di Leo A., Cinque B., Fanini D., Agnifili A., Berloco P., Linsalata M., Lorusso D., Barone M., De Simone C. (2005). Detection of Alkaline Sphingomyelinase Activity in Human Stool: Proposed Role as a New Diagnostic and Prognostic Marker of Colorectal Cancer. Cancer Epidemiol. Biomark. Prev..

[36] Soo I., Madsen K.L., Tejpar Q., Sydora B.C., Sherbaniuk R., Cinque B., di Marzio L., Cifone M.G., Desimone C., Fedorak R.N. (2008). VSL#3 Probiotic Upregulates Intestinal Mucosal Alkaline Sphingomyelinase and Reduces Inflammation. Can. J. Gastroenterol..

[37] Duan R.D., Cheng Y., Jönsson B.A.G., Ohlsson L., Herbst A., Hellström-Westas L., Nilsson Å. (2007). Human Meconium Contains Significant Amounts of Alkaline Sphingomyelinase, Neutral Ceramidase, and Sphingolipid Metabolites. Pediatr. Res..

[38] Nilsson Å., Duan R.D., Ohlsson L. (2021). Digestion and Absorption of Milk Phospholipids in Newborns and Adults. Front. Nutr..

[39] Rankin S.A., Christiansen A., Lee W., Banavara D.S., Lopez-Hernandez A. (2010). Invited Review: The Application of Alkaline Phosphatase Assays for the Validation of Milk Product Pasteurization. J. Dairy Sci..

[40] Clawin-Rädecker I., De Block J., Egger L., Willis C., Da Silva Felicio M.T., Messens W. (2021). The Use of Alkaline Phosphatase and Possible Alternative Testing to Verify Pasteurisation of Raw Milk, Colostrum, Dairy and Colostrum-Based Products. EFSA J.

[41] Marchand S., Merchiers M., Messens W., Coudijzer K., De Block J. (2009). Thermal Inactivation Kinetics of Alkaline Phosphatase in Equine Milk. Int. Dairy J..

[42] Giacometti F., Bardasi L., Merialdi G., Morbarigazzi M., Federici S., Piva S., Serraino A. (2016). Shelf Life of Donkey Milk Subjected to Different Treatment and Storage Conditions. J. Dairy Sci..

[43] Evans L., Crane M., Evans L., Crane M. (2018). The Clinical Companion of the Donkey.

[44] Gur M., Zuckerman-Levin N., Masarweh K., Hanna M., Laghi L., Marazzato M., Levanon S., Hakim F., Bar–Yoseph R., Wilschanski M. (2022). The Effect of Probiotic Administration on Metabolomics and Glucose Metabolism in CF Patients. Pediatr. Pulmonol..

[45] Dieterle F., Ross A., Schlotterbeck G., Senn H. (2006). Probabilistic Quotient Normalization as Robust Method to Account for Dilution of Complex Biological Mixtures. Application In1H NMR Metabonomics. Anal. Chem..

[46] Loidl A., Claus R., Deigner H.P., Hermetter A. (2002). High-Precision Fluorescence Assay for Sphingomyelinase Activity of Isolated Enzymes and Cell Lysates. J. Lipid Res..

[47] Xia Y., Yu J., Miao W., Shuang Q. (2020). A UPLC-Q-TOF-MS-Based Metabolomics Approach for the Evaluation of Fermented Mare’s Milk to Koumiss. Food Chem.

[48] Li M., Kang S., Zheng Y., Shao J., Zhao H., An Y., Cao G., Li Q., Yue X., Yang M. (2020). Comparative Metabolomics Analysis of Donkey Colostrum and Mature Milk Using Ultra-High-Performance Liquid Tandem Chromatography Quadrupole Time-of-Flight Mass Spectrometry. J. Dairy Sci..

[49] Altomonte I., Salari F., Licitra R., Martini M. (2019). Donkey and Human Milk: Insights into Their Compositional Similarities. Int. Dairy J..

[50] Medhammar E., Wijesinha-Bettoni R., Stadlmayr B., Nilsson E., Charrondiere U.R., Burlingame B. (2012). Composition of Milk from Minor Dairy Animals and Buffalo Breeds: A Biodiversity Perspective. J. Sci. Food Agric..

[51] Malacarne M., Criscione A., Franceschi P., Bordonaro S., Formaggioni P., Marletta D., Summer A. (2019). New Insights into Chemical and Mineral Composition of Donkey Milk throughout Nine Months of Lactation. Animals.

[52] O’Callaghan T.F., O’Donovan M., Murphy J.P., Sugrue K., Tobin J.T., McNamara A.E., Yin X., Sundaramoorthy G., Brennan L. (2021). The Bovine Colostrum and Milk Metabolome at the Onset of Lactation as Determined by 1H-NMR. Int. Dairy J..

[53] Lin Y., Sun X., Hou X., Qu B., Gao X., Li Q. (2016). Effects of Glucose on Lactose Synthesis in Mammary Epithelial Cells from Dairy Cow. BMC Vet. Res..

[54] Costa A., Lopez-Villalobos N., Sneddon N.W., Shalloo L., Franzoi M., De Marchi M., Penasa M. (2019). Invited Review: Milk Lactose-Current Status and Future Challenges in Dairy Cattle. J. Dairy Sci..

[55] Arumugam M.K., Paal M.C., Donohue T.M., Ganesan M., Osna N.A., Kharbanda K.K. (2021). Beneficial Effects of Betaine: A Comprehensive Review. Biology.

[56] Shah M., Cabrera-Ghayouri S., Christie L.-A., Held K.S., Viswanath V. (2019). Translational Preclinical Pharmacologic Disease Models for Ophthalmic Drug Development. Pharm. Res..

[57] Holmes-McNary M.Q., Cheng W.L., Mar M.H., Fussell S., Zeisel S.H. (1996). Choline and Choline Esters in Human and Rat Milk and in Infant Formulas. Am. J. Clin. Nutr.

[58] Kim Y.H., Kim D.H., Lim H., Baek D.Y., Shin H.K., Kim J.K. (2009). The Anti-Inflammatory Effects of Methylsulfonylmethane on Lipopolysaccharide-Induced Inflammatory Responses in Murine Macrophages. Biol. Pharm. Bull.

[59] Branco Lopes R., Bernal-Cordoba C., Fausak E.D., Silva-Del-Rio N. (2021). Effect of Prebiotics on Growth and Health of Dairy Calves: A Protocol for a Systematic Review and Meta-Analysis. PLoS ONE.

[60] Nalla K., Manda N.K., Dhillon H.S., Kanade S.R., Rokana N., Hess M., Puniya A.K. (2022). Impact of Probiotics on Dairy Production Efficiency. Front. Microbiol.

[61] Fox P.F. (2015). Book: Chemistry And Biochemistry of Dairy. Handbook of Seafood and Seafood Products Analysis.

[62] Guilloteau P., Martin L., Eeckhaut V., Ducatelle R., Zabielski R., Van Immerseel F. (2010). From the Gut to the Peripheral Tissues: The Multiple Effects of Butyrate. Nutr. Res. Rev..

[63] Meijer K., De Vos P., Priebe M.G. (2010). Butyrate and Other Short-Chain Fatty Acids as Modulators of Immunity: What Relevance for Health?. Curr. Opin. Clin. Nutr. Metab. Care.

[64] Elamin E.E., Masclee A.A., Dekker J., Pieters H.J., Jonkers D.M. (2013). Short-Chain Fatty Acids Activate AMP-Activated Protein Kinase and Ameliorate Ethanol-Induced Intestinal Barrier Dysfunction in Caco-2 Cell Monolayers. J. Nutr..

[65] Liu H., Wang J., He T., Becker S., Zhang G., Li D., Ma X. (2018). Butyrate: A Double-Edged Sword for Health?. Adv. Nutr..

[66] Cant J.P., Kim J.J.M., Cieslar S.R.L., Doelman J. (2018). Symposium Review: Amino Acid Uptake by the Mammary Glands: Where Does the Control Lie?. J. Dairy Sci..

[67] Nemati M., Menatian S., Ghasemi S.J., Hooshmandfar R., Taheri M., Saifi T. (2018). Effect of Protected-Glutamine Supplementation on Performance, Milk Composition and Some Blood Metabolites in Fresh Holstein Cows. Iran. J. Vet. Res..

[68] Newsholme P., Procopio J., Ramos Lima M.M., Pithon-Curi T.C., Curi R. (2003). Glutamine and Glutamate-Their Central Role in Cell Metabolism and Function. Cell Biochem. Funct..

[69] Phang J.M., Liu W., Zabirnyk O. (2010). Proline Metabolism and Microenvironmental Stress. Annu. Rev. Nutr..

[70] Wu J., Liu F., Nilsson Å., Duan R.D. (2004). Pancreatic Trypsin Cleaves Intestinal Alkaline Sphingomyelinase from Mucosa and Enhances the Sphingomyelinase Activity. Am. J. Physiol. Gastrointest Liver Physiol..

[71] Allison M.J., Bryant M.P., Doetsch R.N. (1962). Studies on the Metabolic Function of Branched-Chain Volatile Fatty Acids, Growth Factors for Ruminococci. I. Incorporation of Isovalerate into Leucine. J. Bacteriol..

[72] Vranković L., Aladrović J., Octenjak D., Bijelić D., Cvetnić L., Stojević Z. (2017). Milk Fatty Acid Composition as an Indicator of Energy Status in Holstein Dairy Cows. Arch. Anim. Breed..

[73] Zhang P., Chen Y., Cheng Y., Hertervig E., Ohlsson L., Nilsson Å., Duan R.D. (2014). Alkaline Sphingomyelinase (NPP7) Promotes Cholesterol Absorption by Affecting Sphingomyelin Levels in the Gut: A Study with NPP7 Knockout Mice. Am. J. Physiol. Gastrointest Liver Physiol..

[74] Tsiamita A., Valiakos G., Natsaridis N., Fotiadou S., Manouras A., Malissiova E. (2022). Preliminary Results on the Comparative Evaluation of Alkaline Phosphatase Commercial Tests Efficiency in Non-Cow Milk Pasteurization. BioTech.

